# Neuromodulatory Effects of Alternating Current Electroacupuncture on PTSD-like Behaviors via Gut-Brain Axis Communication

**DOI:** 10.3390/brainsci15121346

**Published:** 2025-12-18

**Authors:** Yijin Jiang, Qixing Wu, Yingjie Liao, Bohan Hu, Fuwen Deng, Hongxu Liu, Shaohui Geng, Guangrui Huang

**Affiliations:** 1 School of Chinese Materia Medica, Beijing University of Chinese Medicine, Beijing 100029, China; 2 School of Life Science, Beijing University of Chinese Medicine, Beijing 100029, Chinagengshaohui@bucm.edu.cn (S.G.); 3 Clinical Medicine College, Chengdu University of Traditional Chinese Medicine, Chengdu 610075, China; 4 School of Nursing, Beijing University of Chinese Medicine, Beijing 100029, China; 5 School of Traditional Chinese Medicine, Beijing University of Chinese Medicine, Beijing 100029, China

**Keywords:** alternating current, electroacupuncture, PTSD, neuroinflammation, gut microbiota, gut–brain axis, TLR4

## Abstract

**Background:** Post-traumatic stress disorder (PTSD) is a debilitating psychiatric condition with limited treatment efficacy. Alternating current electroacupuncture (AC-EA) represents a novel neuromodulatory approach, though its mechanisms—particularly its influence on the gut–brain axis—remain underexplored. **Methods:** We investigated the neurobehavioral and microbiological effects of AC-EA in a rat model of PTSD induced by single prolonged stress. Animals received AC-EA at Baihui (GV20) and Mingmen (GV4) acupoints with varying parameters (0.5 mA/20 Hz, 1 mA/20 Hz, and 1 mA/2 Hz). Behavioral tests (open field test, elevated plus maze), histopathological assessments, immunofluorescence for TLR4, and 16S rRNA sequencing of gut microbiota were performed. **Results:** AC-EA at 1 mA/2 Hz significantly improved exploratory behavior and reduced anxiety-like responses (*p* < 0.05). This regimen also restored neuronal integrity in the hippocampus and cortex and reversed PTSD-induced gut dysbiosis, enriching beneficial genera such as Ligilactobacillus. Furthermore, AC-EA downregulated hepatic TLR4 expression, indicating suppression of neuroinflammatory signaling. **Conclusions:** Our findings demonstrate that AC-EA exerts neuromodulatory and microbiota-rebalancing effects via the gut–brain axis, highlighting its potential as a non-invasive therapeutic strategy for PTSD and related brain health disorders.

## 1. Introduction

Post-traumatic stress disorder (PTSD) is a mental illness that arises from the experience or witnessing of a traumatic event of significant magnitude. The condition’s hallmark symptoms include re-experiencing the event in a traumatic manner, a tendency to avoid related stimuli, a sense of numbing, and heightened vigilance. PTSD exerts a profound impact on the quality of life of affected individuals [1,2,3], while concurrently imposing a substantial economic strain on society [4,5]. Although the current treatment of PTSD relies primarily on psychotherapy and medication, these methods have limited efficacy, high side effects, and poor patient adherence [6]. Consequently, there is a compelling need to investigate safe and effective alternative therapies.

Recent studies have confirmed the potential applications of acupuncture therapy in mental disorders. The therapeutic objectives of acupuncture can be achieved through modulation of nervous system functions [7,8]. Compared with traditional acupuncture, EA features simplified procedures and precisely controllable experimental dosages [9]. By delivering programmed electrical stimulation to specific neural sites via percutaneous needles, EA indirectly affects the conduction of action potential signals [10] and can monitor neural signals or action potential conduction in real time through the use of other neuro-monitoring devices such as EEG [11]. Meanwhile, EA has demonstrated significant efficacy in improving psychiatric symptoms such as anxiety and depression by enhancing treatment tolerance and compliance [12,13,14,15]. Different electrical stimulation modalities exhibit distinct mechanisms and advantages in neural regulation, each influencing brain function through specific pathways. Compared with direct current (DC) stimulation, alternating current (AC) stimulation generates oscillating electric fields in the brain that modulate neuronal firing timing, alter local neural oscillation power, and modify cross-frequency/regional coherence, showing unique advantages in cognitive function improvement [10,16]. In PTSD treatment, frequency-specific AC stimulation can selectively regulate fear memory-related neural circuits by strengthening prefrontal cortex-mediated inhibitory control over the amygdala, thereby facilitating traumatic memory extinction and emotional regulation [17]. Clinical studies indicate that combined EA and AC stimulation significantly alleviates symptom severity in PTSD patients, potentially through mechanisms involving modulation of hypothalamic–pituitary–adrenal (HPA) axis function and promotion of hippocampal/prefrontal cortical neuroplasticity [18,19,20].

The innovative use of AC treatment in this study was guided by the objective of investigating its therapeutic effects and potential mechanisms of action on PTSD-like behavior. In the present study, an experimental model of PTSD in rats was employed to assess its behavioral manifestations. This model was subjected to the open field test (OFT) and the elevated plus maze (EPM), which were used to evaluate the rats’ behavior. The study also investigated the impact of EA on neurons in rats with PTSD. This investigation involved the integration of pathological observation methods, including Nysted staining and immunofluorescence, to explore the effects of EA on neuronal activity in rats with PTSD [17,21,22,23,24]. Furthermore, an examination of the intestinal flora of each group of rats was conducted, in conjunction with combined immunofluorescence experiments, to elucidate the potential mechanism of electrical stimulation for the treatment of PTSD through intestinal flora. The objective of these studies is twofold: first, to establish an experimental basis for the treatment of PTSD by EA, and second, to generate new concepts for the development of therapeutic programs for PTSD.

## 2. Methods

### 2.1. Animals

The experiments were conducted using a total of 28 adult SPF male SD rats, with an average weight of 300 g. Animals were housed under standard laboratory conditions (22 ± 1 °C, 50 ± 10% humidity, and a 12/12 h light/dark cycle). All rats were acclimatized in the animal house for one week. Then, behavioral scoring was performed on each male rat using OFT and EPM to determine the baseline level. The experimental rats were divided into the following groups: High Current and High Frequency AC (AC_H, 1 mA, 20 Hz) group (5 rats), Low Current and High Frequency AC (AC_L, 0.5 mA, 20 Hz) group (5 rats), High Current and Low Frequency AC (AC_A, 1 mA, 2.0 Hz) group (5 rats), control group (4 rats), model group (5 rats), and Positive drug group (10 mg/kg of paroxetine) (4 rats). This division was conducted in accordance with the method of randomized numerical table. No experimental animals, units, or data points were excluded from the analysis in any of the groups. All 28 rats that began the study successfully completed the entire protocol, including modeling, treatment, behavioral tests, and tissue collection. To minimize potential confounders, the order of treatments and behavioral testing was randomized across the different experimental groups each day. Additionally, the cages of all animals were systematically rotated within the housing rack on a regular basis to account for any environmental gradients in the animal room. This study was approved by the Medical and Laboratory Animal Ethics Committee of Beijing University of Chinese Medicine (BUCM-4-2022092902-3118).

### 2.2. Preparation of PTSD Model

**Figure 1:**
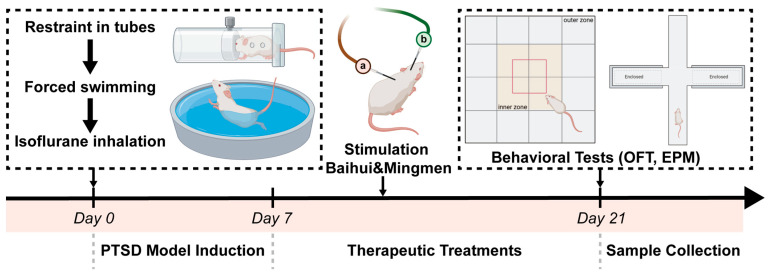
PTSD model preparation, AC treatment, and therapeutic effect validation. a represents Mingmen (GV 4), and b represents Baihui (GV 20).

A rat PTSD model was established using a single prolonged stress method in which rats were confined in plastic tubes (25 cm long and 7 cm in diameter) for 2 h and then immediately forced to swim for 20 min (25 °C). The rats were then allowed to recover from the isoflurane (RWD Life Science Co., Ltd., Shenzhen, China) exposure for 15 min to unconsciousness. The rats were then returned to their cages and kept in a normal, undisturbed environment for 7 days (Figure 1).

### 2.3. Treatment Protocol

Following the completion of the modeling phase, the rats were placed in a fixation cylinder (in an effort to minimize restraint stimulation) and underwent daily EA treatment, commencing on the eighth day. Acupuncture filiform needle (0.35 mm × 25 mm, Suzhou Medical Appliance Factory, Suzhou, China) were applied at the Baihui (GV 20) and Mingmen (GV 4) acupoints. Baihui (GV 20) is located at the midline of the parietal bones. Mingmen (GV 4) is situated on the dorsal midline, in the depression inferior to the spinous process of the second lumbar vertebra. The localization of acupoints was performed in accordance with the standard T/CAAM 0002-2020 “Nomenclature and Location of Acupuncture Points for Laboratory Animals” issued by the Chinese Association of Acupuncture and Moxibustion in 2024. Acupuncture was performed as follows: for Baihui (GV 20), the scalp was pinched up and a filiform needle was inserted horizontally in a posterior direction to a depth of 4–5 mm and then retained; for Mingmen (GV 4), the needle was inserted obliquely at a 45° angle toward the proximal direction to a depth of 2–3 mm and retained. The EA apparatus was connected to an STG4004 stimulator (Multi Channel Systems MCS GmbH, Kusterdingen, Germany). The stimulation parameters were set as follows: waveform = sine wave. Based on prior EA/tACS literature and known frequency-dependent neuromodulatory effects [25], frequencies of 2 Hz and 20 Hz were selected to investigate low- and mid-frequency alternating current neuromodulation, respectively. Current intensities ranging from 0.5 to 1.0 mA were used, which were sufficient to evoke somatosensory responses without causing observable signs of distress. Each treatment lasted for 30 min for a period of 14 days. To eliminate the impact of restraint, the model group was likewise placed within the same immobilization cylinder. Concurrently, the positive group was administered 10 mg/kg of paroxetine (Zhejiang Jianfeng Pharmaceutical Co., Ltd., Jinhua, China) via continuous gavage for a period of 14 days.

### 2.4. Behavioral Tests

The OFT was used to assess psychological stress in rats. The box was 100 cm × 100 cm × 50 cm with black paint around and on the bottom (SA215, Jiangsu Sai’ansi Biotechnology Co., Ltd., Nanjing, China), and the environment was dark. The rats were placed individually in the center of the open field and allowed to freely explore the maze for 5 min. A camera was used to record the rats’ movements in the open field. At the end of the experiment, the rats were removed, the maze was cleaned, evenly sprayed with 75% alcohol (Tianjin Fengchuan Chemical Reagent Co., Ltd., Tianjin, China) and wiped to remove the odor, and finally the data were analyzed using animal behavior software (SANA, Jiangsu Sai’ansi Biotechnology Co., Ltd., Nanjing, China).

The EPM (SA211, Jiangsu Sai’ansi Biotechnology Co., Ltd., Nanjing, China) can be used to assess anxiety responses in rats. At the beginning of the experiment, rats were placed in the maze from the central compartment in the closed direction and activity was recorded for 5 min. At the end of the experiment, the rats were removed, the elevated frame was cleaned, evenly sprayed with 75% alcohol and wiped to remove odor, and finally the data were analyzed using animal behavior tracking system SANS (Jiangsu Sai’ansi Biotechnology Co., Ltd., Nanjing, China).

### 2.5. Histopathological Morphology

On day 21 animals were euthanized by isoflurane overdosage and brains and livers were collected, fixed in 4% paraformaldehyde (Beijing Solarbio Science & Technology Co., Ltd., Beijing, China), embedded in paraffin and sectioned at 5 μm. Hematoxylin and eosin (HE) and Nissl staining were performed using standard protocols.

### 2.6. Immunofluorescence (IF)

Immunofluorescence staining was performed on paraffin sections. Following the fixation of the tissue, the sections were subjected to a standardized protocol involving dehydration, paraffin embedding, and sectioning at a thickness of 5 μm. Prior to staining, the sections were dewaxed with xylene and hydrated with graded ethanol solutions. For antigen retrieval, sections were immersed in a sodium citrate buffer (pH 6.0, Beijing Solarbio Science and Technology Co., Ltd., Beijing, China) and subjected to microwave heating. Subsequently, sections were permeabilized with Phosphate-Buffered Saline (PBS, Wuhan Servicebio Technology Co., Ltd., Wuhan, China) containing 0.1% Triton X-100 (Shanghai Beyotime Biotechnology Co., Ltd., Shanghai, China) and blocked with 5% bovine serum albumin (BSA, Beijing Zhongshan Golden Bridge Biotechnology Co. Ltd., Beijing, China) at room temperature for 1 h. Sections were then subjected to an overnight incubation at 4 °C with a specific primary antibody directed against TLR4 (Abcam #ab22048, 1:100 dilution, Cambridge, UK). Following a thorough PBS wash, the sections were then subjected to an incubation period of one hour at room temperature in the dark, in the presence of the relevant fluorescent secondary antibody (Abcam # ab150117, 1:1000 dilution). Finally, nuclei were counterstained with DAPI (Biorigin Inc., Beijing, China), and sections were sealed with an anti-fluorescence quenching mounting medium. For each liver section, intact hepatic lobule regions were randomly selected as regions of interest (ROIs) for analysis, ensuring structural integrity of the chosen areas. Image processing was performed using ImageJ (version 1.54g). Each ROI was individually binarized using the software’s auto-threshold function to distinguish positive signals from background, with consistent parameters applied across all images. Total fluorescence intensity was quantified using the “Measure” tool. All image analyses were conducted under single-blind conditions: image files were randomly renamed prior to analysis so that the investigator was unaware of the experimental group assignment. Decoding and statistical analysis were performed only after all quantitative data had been collected.

### 2.7. Fecal DNA Extraction and 16S rRNA Sequencing

To validate the initial findings, an independent supplemental cohort (AC_A, Control, and Model groups, *n* = 6 per group) was processed under the same protocol specifically for 16S rRNA sequencing. The data from this validation cohort were analyzed independently and were not pooled with the initial dataset. Fecal samples were collected within a 2 h window (about 08:00) at the end of the active phase (dark cycle) to minimize diurnal variation in gut microbiota. The stools of rats were sent to Major Bioengineering (Shanghai) Co., Ltd. (Shanghai, China) for 16S rRNA sequencing. Total microbial genomic DNA was extracted from stool samples using the Mag Atrract Power Soil Pro DNA Kit (Qiagen, Hilden, Germany) according to manufacturer’s instructions. The quality and concentration of DNA were determined by 1.0% agarose gel electrophoresis and a NanoDrop^®^ ND-2000 spectrophotometer (Thermo Scientific Inc., Waltham, MA, USA) and kept at −80 C prior to further use. The hypervariable region V3-V4 of the bacterial 16S rRNA gene were amplified with primer pairs 338F (5’-ACTCCTACGGGAGGCAGCAG-3’) and 806R (5’-GGACTACHVGGGTWTCTAAT-3’) by an ABI GeneAmp^®^ 9700 PCR thermocycler (ABI, Foster City, CA, USA). The PCR reaction mixture including 4 μL 5 × Fast Pfu buffer, 2 μL2.5 mM dNTPs, 0.8 μL each primer (5 μM), 0.4 μL Fast Pfu polymerase, 10 ng of template DNA, and ddH2O to a final volume of 20 µL. PCR amplification cycling conditions were as follows: initial denaturation at 95 C for 3 min, followed by 29cycles of denaturing at 95 C for 30 s, annealing at 53 C for 30 s and extension at 72 C for 45 s, and single extension at 72 C for 10 min, and end at 4 C. All samples were amplified in triplicate. The PCR product was extracted from 2% agarose gel and purified. Then quantified using Quantus™ Fluorometer (Promega, Madison, WI, USA). Raw paired-end reads were quality-filtered using fastp (version 0.19.6) with the following specific criteria: (i) reads were truncated at any site where the average quality score dropped below 20 over a 50 bp sliding window; (ii) reads shorter than 50 bp after truncation were discarded; (iii) reads containing ambiguous nucleotides (N) were also removed. Subsequently, paired reads were assembled using FLASH (version 1.2.11) with a minimum overlap of 10 bp and a maximum mismatch ratio of 0.2 allowed in the overlap region. Using the UPARSE v11 software (http://drive5.com/uparse/, accessed on 2 May 2025), the quality-controlled and assembled sequences were clustered into operational taxonomic units (OTUs) at a 97% similarity threshold, followed by chimera removal. To ensure equitable comparison across all samples, including those with potentially lower microbial biomass (rare biosamples), all samples were rarefied to an equal number of sequences (33,755 sequences per sample) prior to downstream alpha- and beta-diversity analyses. Taxonomic annotation of the OTUs was performed using the RDP Classifier (version 2.11, https://sourceforge.net/projects/rdp-classifier/, accessed on 2 May 2025) against the SILVA 16S rRNA gene database (v138) with a confidence threshold of 70%. The microbial community composition of each sample was then summarized at different taxonomic levels. Putative functional profiling of the 16S rRNA gene sequences was predicted using PICRUSt2 (version 2.2.0). All bioinformatic analyses were conducted on the Majorbio Cloud Platform (https://www.majorbio.com/).

### 2.8. Statistical Analysis

To minimize bias, the researchers responsible for conducting all behavioral assessments (OFT and EPM) and the subsequent data analysis were blinded to the group identities throughout the experiment. Data analysis was performed using GraphPad Prism (version 9.0). For comparisons across multiple groups (e.g., control, model, positive, AC_L, AC_A, AC_H), one-way analysis of variance (ANOVA) was used, followed by Tukey’s post hoc test for multiple comparisons. A *p*-value of less than 0.05 was considered statistically significant.

## 3. Results

### 3.1. Alternating Current Electroacupuncture Alleviates PTSD-like Behaviors in Rats

**Figure 2:**
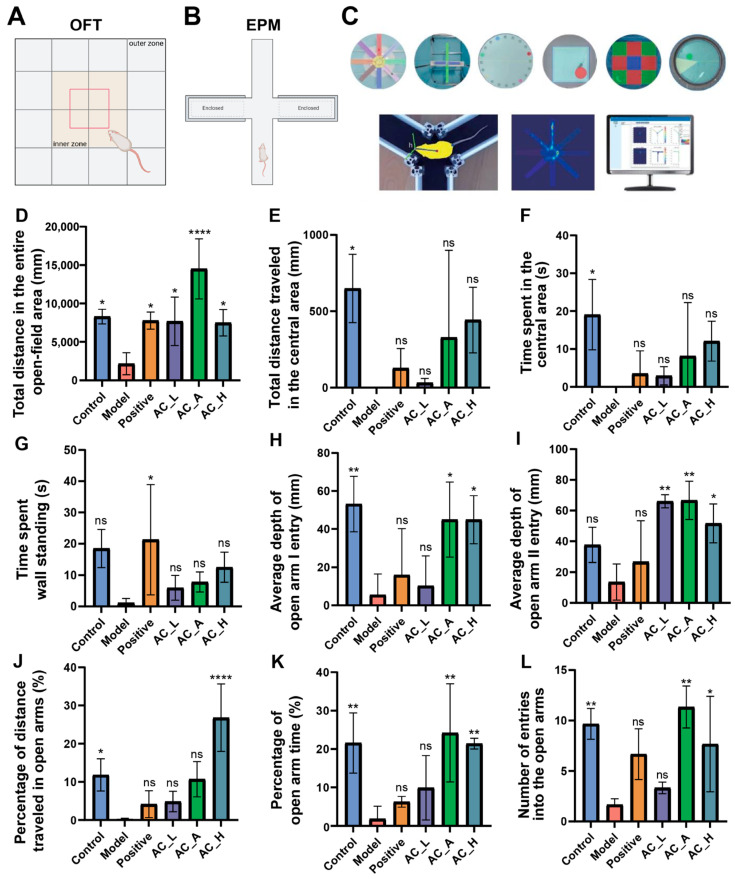
Behavioral tests were performed on rats on day 21. (**A**) Schematic diagram of the rat OFT. (**B**) Schematic diagram of the rat EPM. (**C**) Actual pictures of the elevated rat cross maze. (**D**) Total distance in the entire open-field area of the OFT. (**E**) Total distance traveled in the central area of the OFT. (**F**) Time spent in the central area of the OFT. (**G**) Time spent wall standing of the OFT. (**H**) Average depth of open arm I entry of the EPM. (**I**) Average depth of open arm II entry of the EPM. (**J**) Percentage of distance traveled in open arms of the EPM. (**K**) Percentage of open arm time of the EPM. (**L**) Number of entries into the open arms of the EPM. Group differences were analyzed by one-way ANOVA followed by Tukey’s multiple comparisons test. * *p* < 0.05, ** *p* < 0.01, **** *p* < 0.0001, ns *p* > 0.05 vs. Model group.

The OFT (Figure 2A) was used to assess locomotor activity and exploratory behavior in PTSD model rats, which typically exhibit reduced exploratory drive and spontaneous movement. As shown in Figure 2D, rats in the PTSD model group demonstrated a significant decrease in the total distance traveled in the open field compared with the control group (*p* < 0.05). In contrast, rats in the AC_L, AC_A, and AC_H groups showed a significant increase in total distance traveled compared with the model group (*F* (5, 14) = 7.826, *p* = 0.0011; Model vs. AC_L: *p* = 0.0291; Model vs. AC_A: *p* < 0.0001; Model vs. AC_H: *p* = 0.0381). Although the distance traveled in the central zone, time spent in the center, and wall-standing time in the EA-stimulated groups did not reach statistical significance compared with the model group (*p* > 0.05), these parameters consistently showed a trend toward improvement (Figure 2E–G). These results suggest that AC-EA treatment improves exploratory behavior and spontaneous locomotor activity in rats, effectively ameliorating PTSD-like behaviors. Among the tested parameters, the most pronounced improvement was observed with AC stimulation at 1 mA/2.0 Hz.

Also, EMP (Figure 2B,C) was used to study the psychological state of animals by using their exploratory nature of novel environments and their fear of high-hanging open arms to form contradictory and conflicting behaviors. Compared with the PTSD model group, rats in the AC_A and AC_H groups showed a significant increase in the average entry depth into open arm I (*F*(5, 12) = 5.555, *p* = 0.0071; Model vs. AC_A: *p* = 0.0478; Model vs. AC_H: *p* = 0.0480), whereas the AC_L group exhibited no significant change (Figure 2H). As shown in Figure 2I, the average entry depth into open arm II was significantly greater in the AC_L, AC_A and AC_H groups than in the model group (*F*(5, 12) = 6.360, *p* = 0.0042; Model vs. AC_L: *p* = 0.0040; Model vs. AC_A: *p* = 0.0036; Model vs. AC_H: *p* = 0.0320). Furthermore, PTSD model rats displayed significantly lower percentages of both distance traveled (Figure 2J) and time spent (Figure 2K) in the open arms, as well as fewer open arm entries (Figure 2L), relative to the normal control and several treatment groups. Exploratory trajectories in the open arms visually depicted the spatial exploration patterns of the rats. Trajectories of the PTSD model group were predominantly clustered in the central zone and closed arms, demonstrating typical open-arm avoidance. In contrast, trajectories of the AC_A group extended notably further into the distal sections of the open arms, indicating a significant enhancement of exploratory behavior (Supplementary Figure S1). These findings indicate that AC-EA treatment can enhance exploratory behavior and effectively alleviate PTSD-like behaviors in rats, with the most pronounced improvements observed under AC stimulation at 1 mA/2.0 Hz and 1 mA/20.0 Hz.

### 3.2. Histopathologic Staining Assessment of the Effects of AC on the Brain

**Figure 3:**
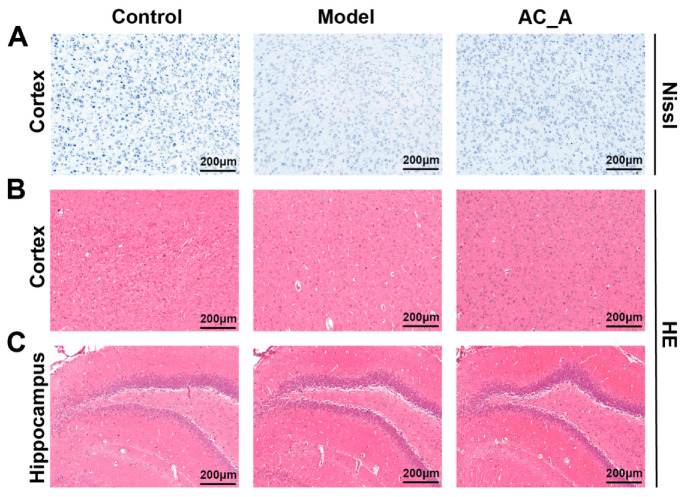
HE and Nissl staining of cortex and hippocampal regions on day 21. (**A**) Nissl staining evaluation of cortex on day 21. (**B**) HE staining evaluation of cortex on day 21. (**C**) HE staining evaluation of hippocampus on day 21.

Nissl staining revealed that the cortical neuronal cells in the Control and AC_A groups exhibited regular arrangement, with dense nuclei and clear nucleoli. The cytoplasm was found to be abundant in Nissl’s body, which exhibited a dark blue coloration. The morphology of the cortical neuronal cells was determined to be abnormal, without the occurrence of neuronal damage loss. The cortical neuronal cells of the model group of rats exhibited abnormalities in their morphology, with most cells displaying signs of rupture. The arrangement of these cells was characterized by sparsity and disorganization, and the number of Nissl’s bodies in the cytoplasm was reduced or disappeared, suggesting a severe degree of neuronal damage (Figure 3A). The number of neurons in the cytoplasm was significantly reduced or disappeared, suggesting that the neurons were severely damaged. In regard to HE staining, as shown in Figure 3B,C, the cytosol of rat CA1 hippocampus and cortical neurons in the Control and AC_A groups exhibited a large, round morphology, accompanied by a light coloration. The nucleoli were similarly large, round, and conspicuous. In contrast, the majority of neurons in the model group exhibited signs of necrosis, with the cytosol displaying wrinkled and deeply stained features, or even dissolution and disappearance. These results indicate that AC can significantly improve the poor morphology of neurons.

### 3.3. AC Modulates Alpha Diversity and Core OTU Composition in PTSD Rats

**Figure 4:**
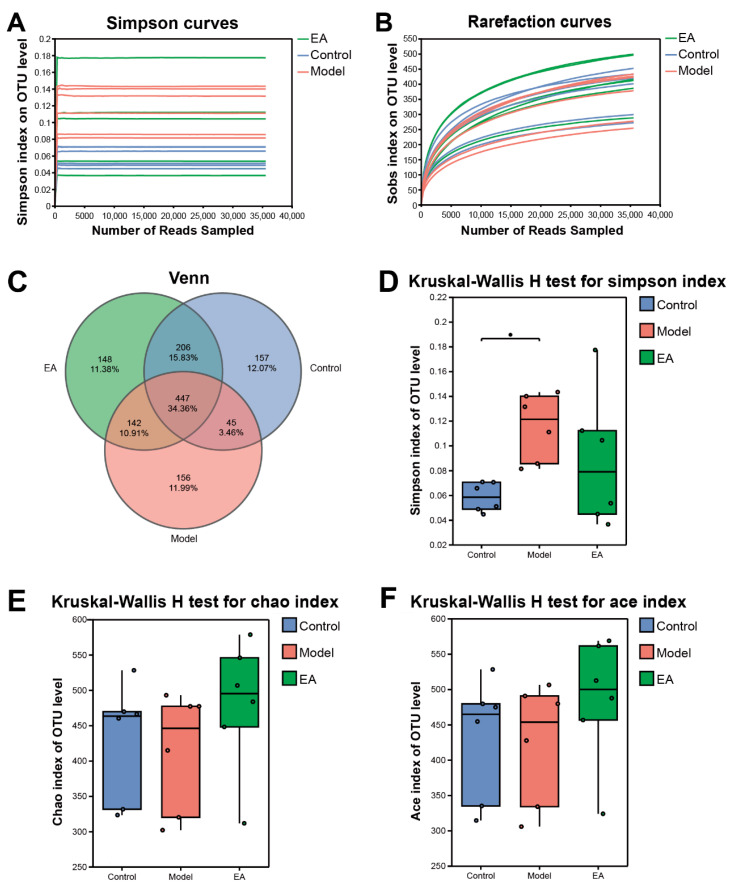
Alpha diversity and core OTU characterization. (**A**) Simpson curve at OTU level. (**B**) Dilution curve based on sobs index. (**C**) Venn graph of common OTUs. (**D**) Simpson index at the OTU level. (**E**) Chao index at the OTU level. (**F**) Ace index at the OTU level.

We investigated the composition of the gut microbiome of Control, Model, EA (AC_A group) rats by 16SrRNA sequencing of fecal samples. After sub-sampling each sample to equal sequencing depth (35,564 reads per sample) and clustering, 6965 operational taxonomic units (OTUs) with 97% similarity were obtained. The number of these OTUs per sample ranged from 272 to 499. The dilution curve can directly reflect the reasonableness of the sequencing data volume, and when the curve tends to be flat, it indicates that the sequencing data volume is asymptotically reasonable. The Simpson curve based on the Simpson index and the dilution curve based on the sobs index (Figure 4A,B) indicate that the sequencing data and depth as well as the sample size are sufficient. As shown in Figure 4C: plotted as a Venn Graph, there were 1301 OTUs common to Control, Model, and EA groups, 157 OTUs specific to the Model group, and 148 OTUs specific to the EA group, suggesting that PTSD and bioelectricity treatments lead to changes in the number of OTUs. Microbial community diversity was analyzed using Alpha Diversity method. Figure 4D showed that compared with the Control group, the Simpson index of the Model group increased significantly, suggesting a trend of increasing species richness in the intestinal flora of PTSD rats. In Figure 4D–F, compared with the Model group, the Chao and Ace indices of the EA group increased and the Simpson index decreased, indicating that AC stimulation altered the microbial community structure.

### 3.4. AC Restructures Microbial Communities and Biomarker Profiles

**Figure 5:**
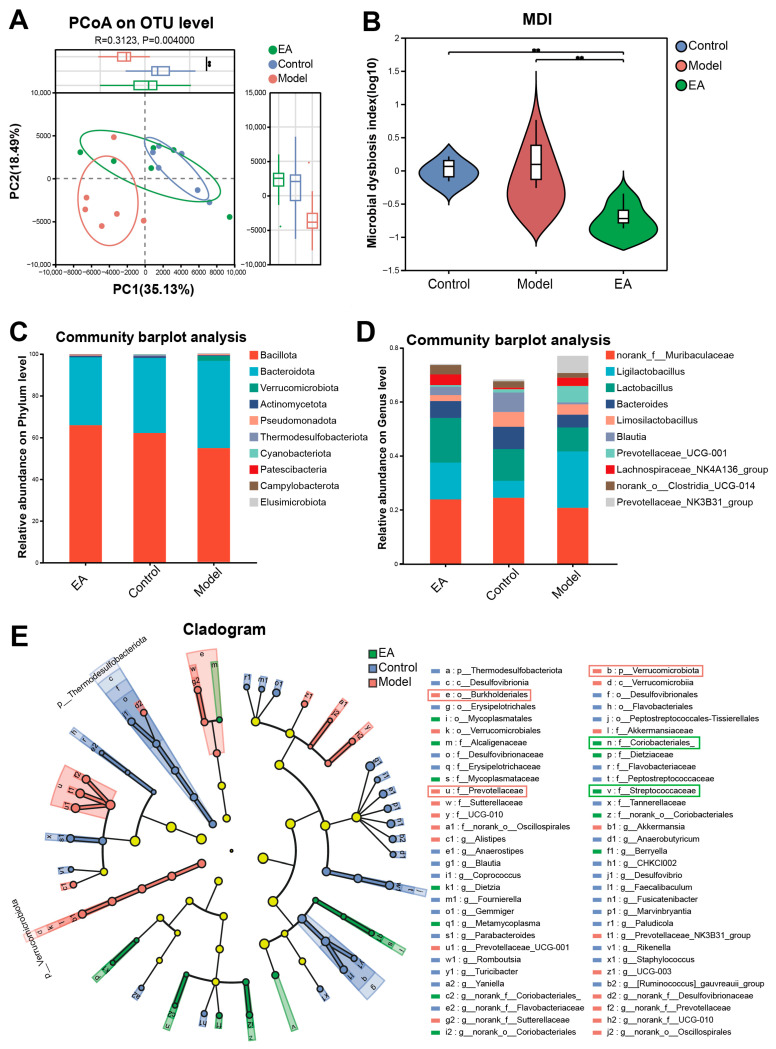
β-diversity, MDI, taxonomy and biomarkers of PTSD gut microbiota. (**A**) PCoA of β-diversity. (**B**) Microbial dysbiosis index (MDI) across groups (OTU level). (**C**) Relative abundance of the top 10 phyla. (**D**) Relative abundance of the top 10 genera. (**E**) LEfSe cladogram of differential taxa (phylum to genus). Representative clado highlighted with boxes: red boxes indicate regions from the model group, and green boxes indicate regions from the EA group. ** *p* < 0.01.

Beta diversity is a measure of differences in species diversity between microbial communities that reveals differences in microbial community structure between samples by comparing species composition between communities. To visualize these differences, the differences between samples can be reflected by principal coordinate analysis (PCoA). In Figure 5A, the distance between samples reflects their similarity in composition and abundance. Specifically, the smaller the distance between the samples, the more similar they are in terms of microbial community composition and species abundance. The Model group was better separated from both Control and EA groups, indicating that PTSD alters the structure of the intestinal flora of rats. And AC stimulation regulates the structural composition and diversity of the intestinal flora of rats with PTSD and has a preventive protective effect.

The intestinal flora dysbiosis index (MDI) is an index that determines the degree of microbial ecological dysbiosis. In Figure 5B, the MDI was lower in the EA group compared to the Control and Model groups, and the results were significant (*p* < 0.01, *p* < 0.01). This suggests that AC stimulation can significantly improve the degree of intestinal flora disorder in rats.

Based on the species annotation results, the top 10 species with maximum abundance at the phylum level were selected for each group to generate a bar chart of species relative abundance (Figure 5C). The dominant phyla in the rat intestine were *Bacillota*, *Bacteroidota*, and *Verrucomicrobiota*. The relative abundance of *Bacteroidota* and *Verrucomicrobiota* in the Model group was elevated, and the relative abundance of *Bacillota* was decreased compared to the Control group. Moreover, compared with the Control group, the relative abundance of *Bacteroidota* and *Verrucomicrobiota* was increased in the Model group and the relative abundance of *Bacillota* was decreased in the EA group.

Based on the results of species annotation, the top 10 species with maximum abundance at the genus level in each group were selected to generate a bar chart of species relative abundance (Figure 5D). Compared with the Control group, the relative abundance of *Bacteroides* in the Model group increased and the relative abundance of *Ligilactobacillus* decreased. After AC stimulation, the relative abundance of *Ligilactobacillus* and *Prevotellaceae*_UCG-001 in the EA group increased, and the relative abundance of *norank_f__ Muribaculaceae*, *Lactobacillus*, *Limosilactobacillus* and *Blautia* decreased.

LEfSe analysis was used to show dominant bacteria in three enterotypic subgroups from the level of Phylum to Genus (Figure 5E, Control, *n* = 31; Model, *n* = 19; EA, *n* = 12). As shown, at the genus level, *Thermodesulfobacteriota* dominates the Control group, and the Model group is dominated by *Verrucomicrobiota*; at the order level, the Model group is dominated by *Burkholderiales* and *Verrucomicrobiia*. At the family level, the Model group was dominated by *Prevotellaceae* and others, while the EA group was dominated by *Coriobacteriales* and *Streptococcaceae*.

### 3.5. AC Reduces TLR4 Expression in Liver

**Figure 6:**
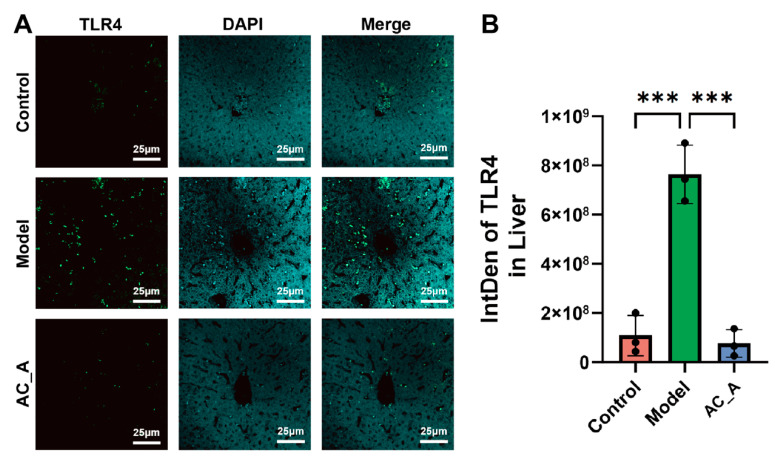
The expression of TLR4 detected by IF. (**A**) IF detection for TLR4 under 40× on day 21. (**B**) IF fluorescence intensity statistics of TLR4 on day 21. *** *p* < 0.001 vs. Model group.

Immunofluorescence analysis (Figure 6) revealed that the fluorescence signal intensity of TLR4 in the liver tissue of rats in the AC_A group was significantly reduced in comparison with the model group. This finding suggests that AC improves PTSD-like behaviors in rats, along with downregulating TLR4 expression and attenuating inflammatory responses in the liver.

## 4. Discussion

This study systematically evaluated the interventional effects of AC-EA at GV20 and GV4 on PTSD-like rats through behavioral assessments, neuronal quantification, gut microbiota analysis, and TLR4 expression profiling. Behavioral results demonstrated that AC electroacupuncture with parameters of 1 mA and 2 Hz significantly improved open-field exploratory behavior and open-arm retention time in the elevated plus maze, while also restoring reduced neuronal counts. Gut microbiota analysis revealed that electroacupuncture intervention altered microbial community structure, reduced the microbial dysbiosis index (MDI), and regulated the abundance of specific bacterial phyla. Furthermore, electroacupuncture significantly decreased hepatic TLR4 expression levels, suggesting its potential to alleviate PTSD symptoms by suppressing inflammatory pathways.

Electroacupuncture is a low-cost and safe intervention [26,27], characterized by rapid efficacy, minimal side effects, and multi-target mechanisms [28,29], providing critical insights for developing novel PTSD therapies. Zhou et al. [30] conducted a systematic review and meta-analysis of electroacupuncture, demonstrating its ability to reduce HAMD scores and recommending its combined use with pharmacological treatments for depression. Hou et al. [31] found that electroacupuncture modulates ventral tegmental area (VTA) inputs to the ventromedial prefrontal cortex (vmPFC), thereby treating PTSD. This study identified 1 mA and 20 Hz as optimal electroacupuncture parameters, which synergistically increased neuronal counts and modulated the gut microbiota-TLR4 axis, unveiling a unique mechanism by which electroacupuncture improves PTSD through gut microbiota-TLR4 regulation. Electroacupuncture significantly increased the abundance of *Bacillota* (encompassing probiotic genera such as *Lactobacillus*) while reducing elevated *Verrucomicrobiota* (associated with intestinal permeability) in the model group. These microbial shifts correlated strongly with behavioral improvements. Additionally, electroacupuncture specifically suppressed hepatic TLR4 expression, delineating a concrete pathway linking gut microbiota alterations to PTSD amelioration.

The gut microbiota, a dynamic ecosystem of symbiotic microorganisms [32], plays a pivotal role in host pathophysiology [33,34,35] through immunomodulation and neuroinflammatory pathways mediated by metabolites [36,37,38,39]. Previous studies have implicated gut microbiota in psychiatric disorders. Xiao et al. [40] employed Mendelian randomization to reveal interactions between host genomes and gut microbiomes that elevate mental disease risks. Muñoz-Pinto et al. [41] demonstrated that gut dysbiosis disrupts ileal mucosal microbial and Th17 immune homeostasis, propagating effects to the brain and exacerbating Parkinson’s disease. Electroacupuncture optimizes gut microbiota structure (e.g., reducing *Bacteroidota* and enriching *Lachnospiraceae*). Current evidence supports the gut microbiota as a pathway for electroacupuncture in treating depression; Wang et al. [42] reported that electroacupuncture alleviates depressive behaviors by modulating gut microbiota and neurotransmitter systems. In this study, PTSD significantly altered the structural and functional composition of rat gut microbial communities. Model rats exhibited abnormally elevated microbial diversity, which electroacupuncture effectively reversed, restoring ecological balance. At the phylum level, PTSD increased pro-inflammatory phyla such as *Bacteroidota* [43] and reduced *Bacillota* (associated with intestinal barrier integrity and anti-inflammatory functions) [44]. Electroacupuncture restored *Bacillota* abundance while suppressing pro-inflammatory phyla. Genus-level analysis revealed PTSD-induced proliferation of endotoxin-associated genera, whereas electroacupuncture enriched probiotic genera with short-chain fatty acid (SCFA)-producing potential. These microbiota changes correlated closely with behavioral improvements. MDI analysis further confirmed that electroacupuncture significantly lowered elevated MDI values in the model group, indicating its role in rebalancing gut homeostasis.

Concurrently, electroacupuncture downregulated hepatic TLR4 expression. TLR4, a key innate immune receptor, activates NF-κB signaling to promote inflammatory cytokine release [45]. Prior studies have shown that lipopolysaccharides (LPS), a major component of Gram-negative bacterial membranes, interact with TLR4 [46], while SCFAs counteract this process [47]. Lai et al. found that the occurrence of PTSD is related to the elevated levels of HMGB1 and TLR4 in the basolateral amygdala. Administration of glycyrrhizic acid (HMGB-1 inhibitor) or LPS-RS (TLR4 antagonist) in the basolateral amygdala can prevent the development of PTSD [48]. Liu et al. found that inflammation mediated by TLR4/MyD88/NF-κ B is involved in the myocardial damage of PTSD [49]. These findings underscore the involvement of TLR4 signaling in both the central nervous system and peripheral organs during PTSD pathogenesis. Our study extends this evidence to the liver, a pivotal metabolic and immunoregulatory organ. The observed modulation of hepatic TLR4 following AC-EA treatment suggests that the gut-liver axis represents a crucial peripheral pathway through which PTSD-associated systemic inflammation can be regulated. Based on the observed TLR4 suppression and gut microbiota remodeling, we propose that electroacupuncture associates microbiota with TLR4 via dual mechanisms: (1) reducing pro-inflammatory bacterial colonization and endotoxin (e.g., LPS) release, thereby attenuating TLR4-mediated inflammation; and (2) modulating microbiota-derived metabolites (e.g., SCFAs) to inhibit TLR4 signaling via portal circulation and vagal afferent pathways, ultimately influencing brain function to alleviate PTSD [50].

Although this study found that AC-EA can downregulate liver TLR4 expression and speculated that it improves PTSD-like behavior by inhibiting TLR4-mediated neuroinflammation, the downstream specific mechanisms of the TLR4 signaling pathway have not been fully elucidated. For example, TLR4 activation can transmit signals through the MyD88-dependent pathway or TRIF-dependent pathway. However, there is still a lack of experimental evidence on whether electroacupuncture selectively inhibits one pathway or affects the cross-talk between the two pathways; the effects of electroacupuncture on downstream phosphorylation events, transcriptional activity, and epigenetic modifications such as NF-κB and MAPK have not been validated in a time gradient or dose-dependent manner. While this study focused on hepatic TLR4 as a key peripheral inflammatory marker, future investigations incorporating direct assessment of TLR4 and inflammatory mediators in relevant brain regions are warranted. In this experiment, in order to control the impact of gender hormone fluctuations on PTSD-related behaviors and neuroendocrine indicators, male rats were selected to construct a PTSD model [51,52,53,54,55,56,57]. In future experiments, male rats and female rats can be included for efficacy and mechanism comparison. This study is based on a rat model, but its clinical translation still faces the following limitations: the equivalent stimulation intensity, frequency, treatment course, and acupoint compatibility of the parameters used in animal studies in humans still need to be optimized, and the animal experiment period is relatively short, which cannot evaluate the long-term efficacy maintenance and potential side effects of electroacupuncture intervention. Clinical translation needs to consider treatment intervals, maintenance cycles, and synergistic effects with other therapies. It should be pointed out that the relatively small sample size in this study may limit statistical testing power. In PTSD models with high behavioral variability, the effect size estimates obtained from small samples may not be stable enough and may be overestimated, which may affect the generalizability of the research results. Therefore, this report should be regarded as a preliminary finding, and its accuracy and generalizability need to be further validated in larger cohorts. In future research, by combining spatial transcriptomics, phosphoproteomics, and metabolomics, the dynamic changes in TLR4 downstream signals under electroacupuncture intervention will be systematically depicted, and the timing of key node changes will be clarified. Phase I/II clinical trials of AC-EA parameters (current, frequency, course of treatment) will be conducted in PTSD patients, and a multicenter randomized double-blind controlled trial will be designed to compare the efficacy and safety of AC-EA with conventional therapies and explore the synergistic effects of combination therapy.

## 5. Conclusions

This study confirms that AC electroacupuncture ameliorates PTSD symptoms by regulating the gut microbiota-TLR4 axis, providing experimental evidence for integrated traditional Chinese and Western medicine. However, limitations remain, including unresolved details of TLR4 downstream signaling (e.g., MyD88, NF-κB) and insufficient clinical translational data. Future research should integrate spatial transcriptomics and metabolic flux analysis to dissect direct microbiota metabolite (e.g., butyrate) regulation of TLR4 pathways and validate electroacupuncture parameters through multicenter clinical trials. The innovation of this work lies in bridging the “gut–brain axis” theory with modern molecular mechanisms, establishing a foundation for noninvasive, multitarget PTSD therapies.

## Data Availability

The raw 16S rRNA gene sequencing data generated in this study have been deposited in NCBI Sequence Read Archive (SRA) under the BioProject accession number PRJNA1358809.

## Supplementary Materials

The following supporting information can be downloaded at: https://www.mdpi.com/article/10.3390/brainsci15121346/s1, Figure S1: Exploratory activity trajectories in the open arms of the EPM.

## Abbreviations

PTSD	Post-traumatic stress disorder
EA	Electroacupuncture
AC-EA	Alternating current electroacupuncture
OFT	Open field test
EPM	Elevated plus maze
DC	Direct current
AC	Alternating current
HPA	Hypothalamic–pituitary–adrenal
HE	Hematoxylin and eosin
IF	Immunofluorescence
OTUs	Operational taxonomic units
PCoA	Principal coordinate analysis
MDI	Microbial dysbiosis index
VTA	Ventral tegmental area
SCFA	Short-chain fatty acid
LPS	Lipopolysaccharides
