# Supplementary material for: Neuromodulatory Effects of Alternating Current Electroacupuncture on PTSD-like Behaviors via Gut-Brain Axis Communication
Source: Brain Sci. 2025 Dec 18;15(12):1346. doi: 10.3390/brainsci15121346 (PMC12730247; doi:10.3390/brainsci15121346)
Supplement: Supplementary file 1 [file brainsci-15-01346-s001.zip › brainsci-4020524-supplementary.pdf]

Supplementary Material

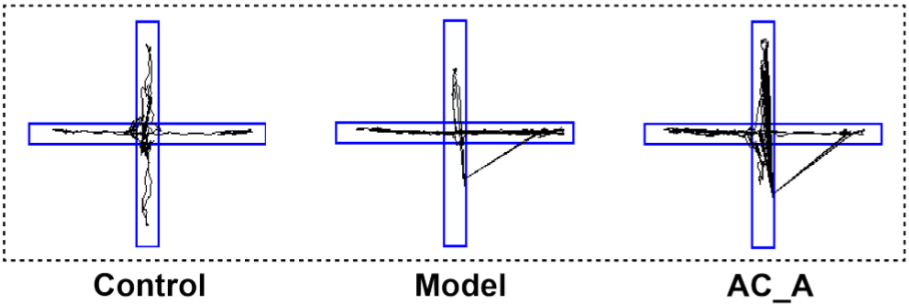

Supplementary Figure S1. Exploratory activity trajectories in the open arms of the EPM.
